# Compound heterozygous *MSH3* germline variants and associated tumor somatic DNA mismatch repair dysfunction

**DOI:** 10.1038/s41698-024-00511-2

**Published:** 2024-01-19

**Authors:** Minoru Koi, Brandie H. Leach, Sarah McGee, Stephanie S. Tseng-Rogenski, Carol A. Burke, John M. Carethers

**Affiliations:** 1 Division of Gastroenterology & Hepatology, Department of Internal Medicine, and Rogel Cancer Center, University of Michigan, Ann Arbor, MI USA; 2 Division of Gastroenterology & Hepatology, Department of Medicine, and Moores Cancer Center, University of California at San Diego, San Diego, CA USA; 3 Center for Personalized Genetic Healthcare, Genomic Medicine Institute, Cleveland Clinic, Cleveland, OH USA; 4 Sanford R. Weiss MD Center for Hereditary Colorectal Neoplasia, Cleveland Clinic, Cleveland, OH USA; 5 Department of Gastroenterology, Hepatology and Nutrition, Cleveland Clinic, Cleveland, OH USA

**Keywords:** Molecular medicine, Cancer genetics

## Abstract

We describe here an individual from a fourth family with germline compound heterozygous *MSH3* germline variants and its observed biological consequences. The patient was initially diagnosed with invasive moderately-differentiated adenocarcinoma of the colon at the age of 43. Germline multigene panel testing revealed a pathogenic variant *MSH3* c.2436-1 G > A and a variant of (initial) uncertain significance *MSH3* c.3265 A > T (p.Lys1089*). Germline genetic testing of family members confirm the variants are in trans with the c.2436-1 G > A variant of paternal and the c.3265 A > T variant of maternal origin. Tumor DNA exhibits low levels of microsatellite instability and elevated microsatellite alterations at selected tetranucleotide repeats (EMAST). Tissue immunohistochemical staining for MSH3 demonstrated variant MSH3 protein is present in the cytoplasm and cell membrane but not in the nucleus of normal and tumor epithelial cells. Furthermore, variant MSH3 is accompanied by loss of nuclear MSH6 and a reduced level of nuclear MSH2 in some tumor cells, suggesting that the variant MSH3 protein may inhibit binding of MSH6 to MSH2.

## Introduction

Compound germline pathogenic variants in the mismatch repair (MMR) gene, *MSH3*, are associated with a colorectal adenomatous polyposis syndrome^1–3^. The incidence of *MSH3*-associated polyposis appears to be extremely rare, and only three families reported to date^1–3^. In the previously reported cases, *MSH3* pathogenic variants led to the complete loss of MSH3 protein from normal colonocytes and tumor cells, resulting in elevated microsatellite alterations at selected tetranucleotide repeats (EMAST) in tumor tissues^1^. Furthermore, these patients exhibited extraintestinal lesions such as gastric cancer, astrocytoma, duodenal adenomas, thyroid adenomas and intraductal papilloma. Here, we describe the 9th case of an individual with germline compound heterozygous *MSH3* variants with early-onset colorectal cancer (CRC). In contrast to previous reported cases, this patient had three adenomatous polyps in addition to his colon cancer and had renal and hepatic cysts as the only extraintestinal lesions. Furthermore, variant MSH3 protein was detected in the cytoplasm and cell membrane in the normal and tumor cells. Associated sporadic nuclear loss of MSH6 protein was also observed in some tumor cells.

## Results

### Case presentation

**Fig. 1:**
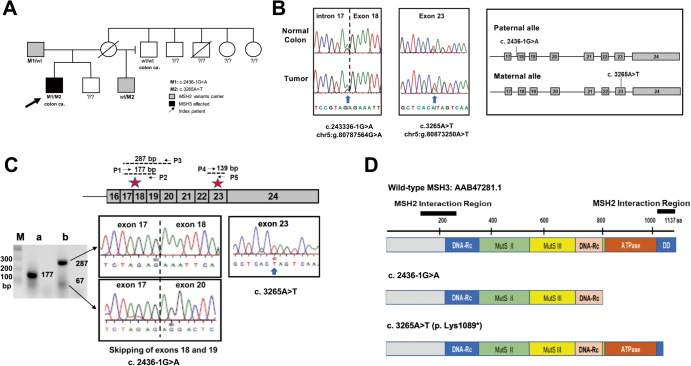
Germline Mutations in *MSH3*. **A** Pedigree of the index patient. Genotype is indicated as combination of *MSH3* M1 and M2 alleles, with M1 as c. 2436-1 G > A and M2 as c. 3265 A > T, and ?/? as unknown. **B** Variants within the *MSH3* gene and location. The variants found through next generation sequencing were validated by Sanger sequencing. The left panel represents chromatograms showing the G to A variant at the splice acceptor site of exon 18 of *MSH3* in normal colon and tumor genomic DNA. The vertical dashed line shows the boundary between intron 17 and exon 18. The arrow indicates G > A variant. The *middle panel* represents chromatograms showing A > T variants in exon 23 (arrow). The right panel shows variant positions within the *MSH3* gene locus. The genomic region spanning from exon 17 to exon 24 of the *MSH3* loci (paternal and maternal) is presented. **C** Variant *MSH3* Transcripts. Top diagram represents partial mature transcript of *MSH3* (from exons 16 to 24). The positions of PCR primers that amplify between exons 17 and 19 (P1/P2), exons 17 and 20 (P1/P3) and within exon 23 (P4/P5) and expected size of each PCR product are presented. The red stars indicate the position affected by the variants. The agarose gel on the left shows the PCR product amplified by P1/P2 (lane *a*) and PCR products amplified by P1/P3 (lane *b*) using cDNA from the index patient. P1/P2 generated a normal 177 bp PCR product indicating that there is no transcript skipping exon 18. P1/P3 generated a normal 287 bp product and abnormal 67 bp products, suggesting that one of two transcripts lost exons 18 and 19. M = molecular marker, in bp. The upper middle chromatogram shows a partial sequence of the 287 bp PCR products that contain normally spliced exons 17 and 18. The bottom middle chromatogram shows a partial sequence of the 67 bp PCR products generated by P1/P3. Skipped exons 18 and 19 were detected due to a paternal c.24336-1 G > A variant. The right chromatogram shows a partial sequence of exon 23 generated by P3/P4 (139 bp). Only variant T due to maternal c.3265 A > T was detected (arrow), indicating that transcript from paternal allele is missing exon 23. **D** Predicted Variant MSH3 Proteins. The top diagram represents wild-type MSH3 proteins (1137aa). The gray region represents the N-terminus of MSH3 containing the NLS and EXO1 interaction region; blue and pink regions contain DNA recognition sites (DNA-Rc); green region has a connector domain; yellow region is the central structure of MSH3; orange region has ATPase activity and a C-terminus; and the blue is the dimerization domain (DD) with MSH2. The black bars represent MSH2 interaction regions. Middle diagram: MSH3 derived from c. 2436-1 G > A is predicted to lose the C-terminus end of 315 aa including ATPase and the DD regions. Lower diagram: MSH3 derived from c 3265 A > T may lose the C-terminus end of 49 aa that is a part of the DD region.

At age 43, our patient presented emergently with an obstructive descending colon cancer requiring urgent laparoscopic left colectomy with end colostomy and Hartmann procedure. Pathology revealed a moderately-differentiated adenocarcinoma that penetrated the surface of the visceral peritoneum (stage IIIB (T4a, N1a, M0). Paired normal (blood) and tumor DNA from the patient was subjected to next generation sequencing (NGS) assay for somatic mutation detection in 648 selected genes (CDx Assay, Tempus, Chicago, IL). Using the IlluminaNovaSeq 6000 platform, hybrid-capture-selected libraries were sequenced at least ≥98% of exons at ≥100× coverage. The patient’s tumor was found to contain somatic mutations in *APC* (p.Q1549*), *APC* (p.R904fs), *SMAD4* (p.R135*), *SMAD4* (p.A190fs), *ATR* (p.F134fs) and *KRAS* (p.G12D). The tumor lacked microsatellite instability (MSI-High) when a PCR-based assay using 5 mononucleotide repeat markers was performed and was not hypermutated by the Tempus test. Immunohistochemical (IHC) staining of the MMR protein PMS2 (as well as MLH1) exhibited nuclear expression in the carcinoma nuclei. In contrast, expression of MSH6 was lost from some carcinoma nuclei. The patient’s blood was subjected to The Common Hereditary Cancers Panel containing 55 genes (Invitae, San Francisco, CA). From this panel only two variants both within *MSH3* were detected: NG_016607.2(NM_002439.5): c.2436-1 G > A (splice acceptor: boundary between intron 17 and exon 18) and NG_016607.2(NM_002439.5): c.3265 A > T (p. Lys1089*, translational stop signal within exon 23) (Fig. 1A–C). The patient’s father, maternal half-brother, and maternal uncle subsequently underwent *MSH3* variant testing. As a result, the *MSH3* c.2436-1 G > A was confirmed to be paternally inherited and the *MSH3* c.3265 A > T (p. Lys1089*) was confirmed to be maternally inherited (Fig. 1A).

Six months after partial colectomy and adjuvant chemotherapy, a colonoscopy revealed two 5 mm tubular adenomas at the hepatic flexure and transverse colon. Metastatic disease to the liver was observed 17 months after the initial colon resection, and a trial of pembrolizumab was given. Colonoscopy performed 2 and 4 years after surgery demonstrated an additional 5 mm tubular adenoma in the distal transverse colon and one hyperplastic polyp, respectively. His metastatic disease continues to progress despite additional chemotherapy.

### Characterization of MSH3 mutations

Both germline *MSH3* variants were confirmed by Sanger sequencing of the PCR product generated from patient’s normal colon and tumor tissue DNA (Fig. 1B). To detect a transcript from each of the variant loci, mRNA was isolated from the formalin-fixed and paraffin-embedded (FFPE) tumor tissues, and reverse transcribed. Because the c.2436-1 G > A variant was located at the splicing acceptor site of the coding exon 18 (Fig. 1B, C), we speculated that transcripts might skip exon 18 and/or other down-stream exons. To capture the predicted transcripts where exon 18, or 18 and 19 were skipped, a 177 bp cDNA segment was amplified by primer sets P1 and P2 (P1/P2), and a 287 bp segment was amplified by primer sets P1 and P3 (P1/P3) (Fig. 1C, top diagram). As shown in the agarose gel electrophoresis image in Fig. 1C, a single 177 bp PCR product generated by P1/P2 primers (Lane a) appeared, indicating that there was no transcript with skipped exon 18. On the other hand, primers P1/P3 generated 2 main PCR products (67 bp and 287 bp) (Lane b). These PCR products were cloned into plasmid vectors and sequenced. The 287 bp segment contained a normal splicing product between exons 17 and 18 whereas the 67 bp segment exhibited a splicing product between exons 17 and 20, which skipped exons 18 and 19 (Fig. 1C). These results indicate that the *MSH3* transcript from the maternal allele may contain intact exons 17, 18, 19 and 20, whereas the *MSH3* transcript from the paternal allele lost the exon 18 and 19 sequences. To detect the maternal transcript with the c.3265 A > T variant in exon 23, a cDNA segment spanning exon 23 was amplified by primer sets P4 and P5 (P4/P5) (Fig. 1C, top diagram), then subjected to TA cloning and sequenced. Ten out of ten plasmid clones exhibited variant c.3265 T but not wild-type c.3265 A (Fig. 1C), suggesting that the paternal transcript may not contain exon 23. Because a change from A to T at c.3265 generates a stop codon, protein encoded by the maternal allele should be truncated at p.1089 (Fig. 1D). Taken together, the observed genomic variants result in the expression of mutated *MSH3* transcripts. The effects of c.2436-1 G > A were revealed as transcript skipping exons 18 and 19, creating a terminal codon within exon 20. The resulting protein may consist of 817 amino acids (aa) that has lost the ATPase and one of the dimerization domains (DD) with MSH2 (Fig. 1D). However, there is also a possibility that the transcript from the c.2436-1 G > A allele may have undergone nonsense-mediated mRNA decay. The effects of c.3265 A > T results in a premature translational stop signal in the *MSH3* gene (p. Lys1089*), generating a truncated protein that may lose 49 aa within the protein’s DD domain (Fig. 1D).

### MSI/EMAST in tumor tissue

**Fig. 2:**
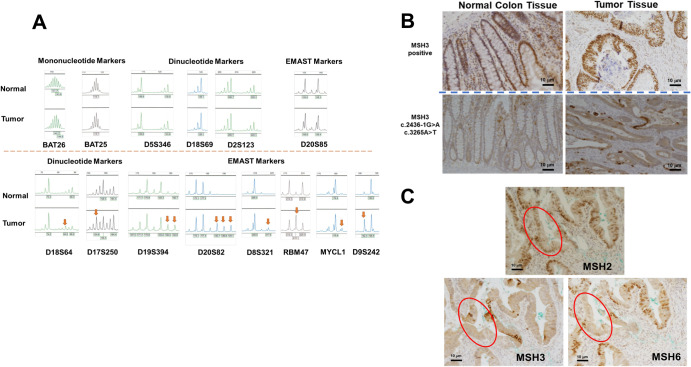
Variant MSH3 expression and its consequences in tumor tissues. **A** Microsatellite instability and EMAST in normal colon and tumor tissues. Two markers with mononucleotide repeats, 5 markers with dinucleotide repeats, and 7 EMAST markers were tested for instability in tumor DNA compared to normal colon DNA. Red arrows indicate new alleles found within tumor DNA. **B** IHC staining of MSH3 in normal colon and tumor tissues. The upper left and right images show normal colon and tumor tissue, respectively, that are positive for MSH3 IHC staining. The bottom left and right images show normal colon and tumor tissues from the index patient. Note that MSH3 is present in the cytoplasm and cell membrane in both the normal colon and tumor cells. Intense staining of MSH3 is seen in the cell membrane of some tumor cells. Scale bars are 10 micrometers. **C** Loss of MSH6 and reduced MSH2 in the patient’s *MSH3* variant tumor cells. The consecutive FFPE slides were IHC stained with anti-MSH2, anti-MSH6, and anti-MSH3 antibodies. In some but not all tumor glands, simultaneous loss of MSH6 and reduced MSH2 was observed (*areas circled in red*). Scale bars are 10 micrometers.

To determine functionality of the observed compound heterozygous *MSH3* variants in tumor vs normal tissue, we examined instability of the microsatellites with mono- and dinucleotide repeats and that of elevated microsatellite alterations at selected tetranucleotide repeats (EMAST) markers^4,5^. The results showed that none of the microsatellites with mononucleotide repeats exhibited frameshift mutations when compared to normal tissue, while two of the five markers with dinucleotide repeats and six of the seven EMAST markers exhibited instability (Fig. 2A). These results demonstrated that the MMR function of MSH3 protein had been completely lost within the patient’s tumor^6,7^.

### Cytoplasmic and membranous expression of variant MSH3 and loss of MSH6 in tumor nuclei

Using a rabbit monoclonal anti-MSH3 antibody (clone 405) we detected nuclear MSH3 protein expression in the non-patient control normal crypts and CRC tumors by IHC, whereas this patient’ MSH3 variant proteins were detected in the cytoplasm and/or cell membrane but not in the nucleus of either the patient’s tumor or normal colon tissues (Fig. 2B). Strong cytoplasmic and membrane-bound MSH3 signals were detected in some cells in both the tumor and normal colon tissues, suggesting that variant MSH3 accumulates in cytoplasm/membrane. Because MSH3 shares MSH2 as a heterodimer partner with MSH6, variant MSH3 protein might affect relative levels and functionality of MSH6 and MutSα (the heterodimer of MSH2 with MSH6). We, therefore examined the cellular protein expression of MSH2 and MSH6 using IHC. Surprisingly, we detected reduced expression of MSH2 and loss of MSH6 expression in some of the tumor cells, suggesting that variant MSH3 protein may influence the stability or availability of MSH2 and MSH6 (Fig. 2C). These results suggest that transcripts encoded by either or both *MSH3* locus with c.2436-1 G > A and c.3265 > T variants may be translated as truncated MSH3 protein(s), may not be recruited to the nucleus, and become sequestered in the cytoplasm/cell membrane. The results also suggest that variant MSH3 protein may affect heterodimer formation between MSH2 and MSH6.

## Discussion

To date, three families affected by *MSH3* germline compound heterozygous variants, with a total of six pathogenic variants, have been reported^1,2^. These variants are predicted to result in loss of MSH3 function. MSH3 encoded by four of the variants, including c.1148delA, c.2319-1 G > A, c.2760delC, and c.3001-2AC, was undetectable in either the nucleus or cytoplasm of normal colon and tumor cells by IHC staining^1^. Transcripts of two other variants, c.2409 C > A and c. (1340 + 1_1341-1) (2655 + 1_2656-1) del, are predicted to have undergone nonsense-mediated mRNA decay^2^.

Here, we report 2 additional variants of *MSH3*, c.2436-1 G > A (CRCh38: chr5: g. 80787564 G > A) and c.3265 A > T (CRCh38: chr5: g. 80873250 A > T), that are likely pathogenic.

However, compared to the other patients/families reported^1,2^, the number of polyps in this patient is small (three small tubular adenomas and one hyperplastic polyp within 4 years after colon cancer diagnosis), and no reported extraintestinal lesions^1,2^ were found with the exception of small bilateral renal cysts and hepatic cysts. It is not known whether this is due to the specific nature of the *MSH3* variants or if some additional factor is necessary for the onset of polyposis.

We predicted the secondary structures of each variant MSH3 protein based on the expressed transcripts (Fig. 1D). As shown by MSH3 IHC staining where the anti-MSH3 antibody utilized recognizes the N-terminus of MSH3, either one or both mutated proteins are translated and remain in the cytoplasm but not in the nucleus of normal colon and tumor cells and seem to accumulate in the cytoplasm and the cell membrane of several tumor epithelial cells (Fig. 2B). The reason for the heterogenous presence of variant MSH3 among colon cells is not clear. However, compared to wild-type MSH3 that is susceptible for degradation without MSH2, variant MSH3 is resistant to degradation even without MSH2 (Fig. 2C), suggesting that variant MSH3 may gradually accumulate in cytoplasm/cell membrane. It could be that cells with higher levels of variant MSH3 may experience cell death and be eliminated from the tissues. Loss of nuclear MSH3 explains the observed genetic instability, MSI-L/EMAST, found enhanced in tumor tissues (Fig. 2A). The results also indicate that the variant MSH3 must have lost its nuclear localization function. We recently identified the MSH3 region responsible for generating the nuclear localization signal (NLS) (aa 84–107)^8^. Because the predicted MSH3 from both mutations retains this region (Fig. 1D), conformational changes due to the variants may hinder the functional NLS.

A surprising finding from our analysis is that sporadic loss of nuclear MSH6 and simultaneous reduction in nuclear MSH2 were present in some tumor cells (Fig. 2C). Considering that truncated MSH3, either from c.2436-1 G > A and/or c.3265 A > T, retains one of the MSH2 interaction regions (135-259)^9^ (Fig. 1D), one explanation for this phenomenon would be that variant MSH3 protein may compete with MSH6 for MSH2 binding, reducing availability of MSH2 and reducing stability of existing and newly synthesized MSH6, as these proteins require heterodimerization for stability^10^. Because the presence of variant MSH3 in the cytoplasm or membrane is not apparently accompanied by presence of MSH2, binding between variant MSH3 and MSH2 may be transient, and the MSH3 variant protein might be resistant to degradation as an individual, non-MSH2 bound protein as discussed above. This can be explained by the loss of another MSH2 interaction region (1065-1137) in the variant MSH3 protein^9,11^ (Fig. 1D). Reduction of normal MSH6 in the cytoplasm and/or nucleus, and abnormal MSH3 in the cytoplasm may result in destabilization of MSH2. Although the NGS test did not either detect somatic mutations in the *MSH6* locus even more than 100X coverage or characterized this tumor as hypermutated (tumor mutation burden was 6.8 mutations per mega base), another explanation for the loss of MSH6 could be that a small number of tumor cells carry isolated somatic mutations in the *MSH6* genes. However, this is not likely because MSH6-negative cells and MSH6-positive cells exist in a mosaic fashion in a single gland (Fig. 2C), it is hard to imagine all MSH6-negative cells are derived from the same single mutant cell. It is also hard to imagine MSH6-negative cells are derived from more than one mutated stem cell. Further studies are needed to determine whether loss of MSH6 in some tumor cells is mechanistically related to the expression of variant MSH3 or not.

Because tumor cells that lost MSH6 generally exhibit high mutation rates in many genes and high levels of microsatellite instability, minority portions of this patient’s tumor could be a mixture of EMAST plus MSI-H and in theory could benefit treatment with combined use of some types of chemotherapy^12^ and immune checkpoint inhibitors^13^ for which was tried as therapy for this patient.

It remains to be determined whether both or one of the two variants is responsible for the expression of the abnormal MSH3 protein. An approach would be to generate colon cell lines containing each of the 2 variants via CRISPR/Cas9 gene editing to further determine biological effects of these mutations on the mismatch repair system and mutation burden. Such an approach could also help determine whether and how variant MSH3 contributes to adenoma formation.

In conclusion, this study describes the ninth known patient with biallelic *MSH3* germline variants that are pathogenic and associated with colorectal adenomas and carcinoma. The discovery of this patient was through multigene panel testing based on his early-onset colon cancer diagnosis with lack of MSI-H in the tumor despite partial loss of MSH6 expression. This patient did not present with polyposis and suggests that there may be additional cases with biallelic pathogenic *MSH3* germline variants without severe polyposis as a clinical presentation, and MSH3 analysis should be considered in all individuals with early-onset colorectal cancer.

## Methods

### Use of patient information

This study was approved by Institutional Review Boards at both the Cleveland Clinic Foundation and the University of Michigan. All authors have complied with all relevant ethical regulations including the Declaration of Helsinki. Informed consent was obtained for this patient and is maintained on file at the Cleveland Clinic Foundation.

### Isolation of genomic DNA and mutation detection

Formalin-fixed and paraffin-embedded (FFPF) normal colon or tumor tissues was micro-dissected and subjected to DNA isolation using a commercially available kit (Quagen). To detect the c.2436-1 G > A mutation, a genomic DNA segment spanning the boundary between intron 17 and exon 18 of *MSH3* was amplified by PCR using the following primers: forward: CTGTTAGGAATGCCTGCTGAT; reverse: CTCCTTGCTTAGCGACCTTG. PCR products were cloned into a plasmid vector by TA cloning and subjected to Sanger sequencing. To detect the c.3265 A > T mutation, a DNA segment containing exon 23 of *MSH3* was amplified using the following PCR primers: forward: CAGCAGAACAAGTCCCTGAT; reverse: TAGGACGCTCCTCTCTTTCT. The PCR products were cloned into a plasmid vector and sequenced.

### Isolation of mRNA and detection of MSH3 transcripts

RNA was isolated from micro-dissected FFPF-tumor tissues and reverse transcribed. The resulting cDNA was used as a template for detecting transcripts from each of the mutated *MSH3* loci. To detect transcripts affected by the c.2436-1 G > A mutation, cDNA segments spanning from exon 17 to 19 for exon 18 skipping and one spanning from exon 17 to 20 for exons 18 and 19 skipping were PCR-amplified, TA cloned and sequenced. The following PCR primers were used for exon 18 skipping detection: forward P1: CAGTGCTGAATGGCTTGATT; reverse P2: CTTTCTTCTTGTACAGTTGG. For exons 18 and 19 skipping detection: forward P1: CAGTGCTGAATGGCTTGATT; reverse P3: TGTTTGGTCCGGTAATTATC. To detect transcripts affected by the c.3265 A > T mutation, a cDNA segment containing exon 23 was PCR-amplified, TA-cloned and sequenced. The PCR primers used were as follows: forward P4: GGAGTTATGGATTAAATGTGGC; reverse primer P5: CTTGAGTGTCTTTCTTTTCG.

### MSI/EMAST assay

The MSI/EMAST assay was previously described^4^. Briefly, normal- and tumor-derived DNA was subjected to 4 separate multiplex PCR amplification (a total of 14 microsatellite markers) using a QIAGEN Multiplex PCR Kit (QIAGEN, Hilden, Germany). These markers include 2 mononucleotide (*BAT25* and *BAT26*), 5 dinucleotide (*D2S123*, *D5S346*, *D17S250*, *D18S64*, and *D18S69*), and 7 tetranucleotide microsatellite markers (*D9S242*, *D20S82*, *D20S85*, *D19S394*, *D8S321*, *MYCL1*, and *RBM47*. We defined EMAST as at least 1 tetranucleotide marker with frameshift without any frameshift of a mononucleotide marker and combined this group with MSI-L samples due to both MSI-L and EMAST being caused by MSH3 dysfunction^5,7^.

### Immunohistochemical staining

Five-micrometer thicknesses of FFPE-tumor and normal colon tissues on slides were deparaffinized with xylene and ethanol washes, followed by heating in Tris-EDTA buffer (pH 9.0) at 121 °C. Samples were then processed with endogenous peroxidase quenching and blocked with 2.5% horse serum (ImmPRESS Universal PLUS Polymer Kit; Vector Laboratories). Primary antibody staining was performed with rabbit monoclonal MSH2 antibody (RevMAb Biosciences, clone RM375, at a dilution of 1:100), rabbit monoclonal MSH6 antibody (BETHYL, clone BLR117H at 1:100) or rabbit monoclonal MSH3 antibody (RevMAb Biosciences, clone 405, at 1:200) overnight at 4 °C. Samples were washed and incubated with a secondary antibody (ImmPRESS-HRP universal polymer reagent) for 1 hour at room temperature and were developed with 3,3′-diaminobenzidine, followed by counterstaining with hematoxylin. The stained tissues were observed through an inverted microscope (ix71, Olympus, PA) using x 20 objective lens. The images were captured by digital camera (DP74, Olympus) and were further stored using image software (cellSens, Olympus). The stored images were reproduced and processed using adobe photoshop (San Jose, CA).

### Reporting summary

Further information on research design is available in the Nature Research Reporting Summary linked to this article.

### Supplementary information

Reporting Summary

## Data Availability

Data from this study is available upon request to the corresponding author. Invitae has submitted both *MSH3* variants to ClinVar https://www.ncbi.nlm.nih.gov/clinvar/variation/655023/ and https://www.ncbi.nlm.nih.gov/clinvar/variation/951203/: NM_002439.5(MSH3):c.2436-1 G > A, Accession: VCV000655023.10, Variation ID: 655023 and NM_002439.5(MSH3):3265 A > T(p.Lys1089Ter), Accession: VCV000951203.4, Variation ID: 951203.
